# Controlling the motion of gas-lubricated adhesive disks using multiple vibration sources

**DOI:** 10.3389/frobt.2023.1231976

**Published:** 2023-10-30

**Authors:** Chengzhe Jia, Sankaran Ramanarayanan, Antonio L. Sanchez, Michael T. Tolley

**Affiliations:** ^1^ Department of Mechanical and Aerospace Engineering, University of California San Diego, La Jolla, CA, United States; ^2^ Materials Science and Engineering Program, University of California San Diego, La Jolla, CA, United States

**Keywords:** vibration, adhesion method, robotic, gas-lubricated, torque generation, locomotion

## Abstract

Robots capable of generating adhesion forces that can achieve free movement in application environments while overcoming their own gravity are a subject of interest for researchers. A robot with controllable adhesion could be useful in many engineered systems. Materials processing equipment, robots that climb walls, and pick-and-place machines are some examples. However, most adhesion methods either require a large energy supply system or are limited by the properties of the contact plane. For example, electromagnetic adhesion requires a ferromagnetic surface and pneumatic adhesion requires a flat surface. Furthermore, nearly all existing approaches are only used to generate adhesion forces and often require additional mechanisms to remove the adhesive component from the surface. In this study, we aimed to develop a simpler method of adhering to a surface while simultaneously moving in directions parallel to the surface, using multiple vibration sources to generate normal adhesion and propulsion. To test our approach, we constructed circular and elliptical models and conducted experiments with various inputs and model parameters. Our results show that such a gas-lubricated adhesive disk could achieve adhesive rotation and displacement in the plane without requiring any auxiliary operating system. Using only vibration sources, we were able to generate the necessary adhesion and propulsion forces to achieve the desired motion of the robot. This work represents a step towards the construction of a small-sized tetherless robot that can overcome gravity and move freely in a general environment.

## 1 Introduction

Robots that can move freely in a designated space, even against gravity, have long been a goal of researchers’ efforts. Yet, locomotion on steep slopes or inverted surfaces is a challenge for mobile robots. Most of the traditional movement methods rely on gravity to achieve movement with the driving force in the horizontal direction. However, when the robot needs to climb up a steep slope or move on inverted surfaces, we need an additional adhesion force to ensure the robot is always attached to the surface. A robot with controllable adhesion could be useful in many engineered systems (Xu et al., 2022). Materials processing equipment (Li and Wong, 2006), robots that climb walls (Pretto et al., 2008; Nansai and Mohan, 2016), and pick-and-place machines (Koepp et al., 2008) are some examples. The controllable adhesion of robots makes them ideal for inspection (Song et al., 2008; Brusell et al., 2016), monitoring (Dunbabin and Marques, 2012), and exploration of environments not suitable for humans (Murphy and Sitti, 2007).

Many techniques have been developed for controllable adhesion (Sikdar et al., 2022; Fang and Cheng, 2023), based on fibrillar (Murphy et al., 2011), pneumatic (Lee et al., 2015), and electromagnetic (Grieco et al., 1998) adhesive forces. These techniques have their own advantages and disadvantages. Pneumatic adhesion (Shi and Li, 2020) and electromagnetic adhesion (Zhang et al., 2021) methods have been used to generate high adhesive forces and allow robots with a heavy load to climb up walls (Pretto et al., 2014). Some robots have been designed to be weakly contacted so they could slide along the adhering surface with low or even no friction (Xiao and Wang, 2015). However, all methods work on a limited range of surfaces: the pneumatic adhesion method requires a flat surface and the electromagnetic adhesion method requires a ferromagnetic surface. The design of these robots requires extra hardware like pumps and magnets. The power requirements are also issues in robot design. To make a robot without an external energy supply, the size of the robot would need to be increased to carry a high-voltage power source or high-pressure air pump. As for the dry fibrillar method, using patterned arrays to induce Van der Waals force could provide considerable strength-to-weight performance and could stay attached without consuming power. But fibrillar adhesives must be designed for a specific range of load angles and have high demands on the cleanliness of the plane (Kim et al., 2007; Marvi et al., 2015). Moreover, all the listed methods use the adhesion force only for sticking to a surface, while a separate type of actuation is still required to achieve motion along the surface.

**FIGURE 1:**
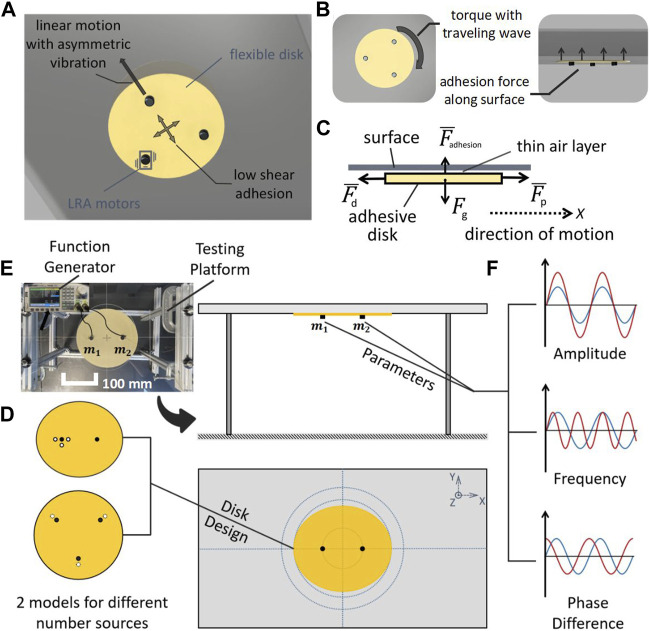
**(A)** Schematic of vibration adhesion disk robot on a surface in isometric view, **(B)** bottom view, and side view. **(C)** Free-body diagram of the robotic disk, with bars indicating time-averaged values. **(D)** Schematic of two experimental models: an elliptical disk with two vibration sources, and a round disk with three vibration sources. **(E)** Real and schematic of the experimental setup for testing the robotic disks. (Scale bar denotes 100 mm). **(F)** Parameters for varying input signals to the vibration sources.

Vibration-based adhesion is another method used to generate an effective adhesion force. Such a method has many unique advantages and features which have not been extensively studied. However, many publications have explored a technique called squeeze-film (or near-field acoustic) levitation (SFL) over the past few decades, which uses vibration to achieve suspension, rotation and transportation of objects without physical contact (Wei et al., 2018; Gabai et al., 2019). In conventional SFL, a disk is held near a parallel wall and oscillated along its surface-normal axis by a centrally-mounted, high-frequency vibration source. The disk oscillations give rise to oscillatory airflow in the thin air layer confined between the disk and the wall (see Figure 1C). Due to the inherently nonlinear dynamics of this unsteady, viscous, compressible flow, a time-averaged pressure is generated within the air layer, that is, in general, unequal to the ambient pressure experienced by the opposite surface of the disk. If the disk is sufficiently flexible, pressure variations in the air layer may non-negligibly affect its dynamics, as seen in our previous work (Weston-Dawkes et al., 2021), resulting in two-way fluid-structure coupling. Based on operating parameters such as the oscillation frequency, disk radius, characteristic thickness of the air layer, and disk oscillation waveform, the steady pressure force experienced by the disk may act to repel it from, or attract it toward, the wall, allowing repulsive or adhesive levitation. Previous theoretical studies have predicted that highly flexible oscillators allow the generation of adhesive forces significantly larger than those produced using stiffer oscillators (Ramanarayanan et al., 2022; Ramanarayanan and Sánchez, 2022). This has been confirmed in experiments where thin plates were oscillated using sound exciters, allowing suspension of heavy loads beneath a flat surface (Colasante, 2015).

In a previous work, we exploited this method to achieve robotic transport. By using a flexible disk and a single ERM (Eccentric Rotating Mass) vibration motor, we generated a strong and controllable attraction force with commercially available hardware (Weston-Dawkes et al., 2021). The robot demonstrated locomotion on a curved surface, vertical climbing, and movement on an inverted surface. We installed two wheels on the bottom of the disk to produce the force and torque we needed in directions parallel to the adhering surface. However, such a design required a suspension system to generate a normal force, effectively reducing the adhesion force and diminishing the maximum load. Moreover, when using differential drive steering to rotate the mobile system on the curved surface, the adhesion failed. This motivated us to extend our previous work to study the possibility of generating torque and locomotion forces without reliance on a conventional drivetrain.

A recent theoretical study claims that asymmetrical flexural oscillations of a flat plate can be used to achieve thrust forces with adhesive levitation (Ramanarayanan and Sánchez, 2023), but to the best of our knowledge, there has yet been no experimental proof of this proposition. In this paper, we demonstrate, for the first time, the ability of an oscillated robotic disk to achieve adhesion to a flat surface while generating linear movement and rotation with low air resistance (see in Figures 1A, B). The required lateral forces and moments are generated by inducing traveling-wave deformations of the disk using multiple vibration sources, eliminating the need for physical contact with the surface.

## 2 Methods

### 2.1 Experimental setup

The experimental equipment comprised three components: a disk-shaped robot, a function generator that produced the modulation and energy signals, and a test platform for measuring and supporting the robot’s operation.

#### 2.1.1 Adhesive disk design

We used flexible plastic, vibration motors, and copper wire to build the vibration adhesion disk. The disks were made of 0.004” Polyester Plastic (color Beige, McMaster-Carr), which was cut into a specified shape (circle or ellipse) using a laser cutter and engraved with the locations of the vibration sources. We chose Linear Resonant Actuators (LRA) motors (model VG1040003D, Vybronics) as the vibration sources, with a rated input voltage range of 0.1 V–2.5 V and an input frequency range of 150 Hz–200 Hz. The LRA motors have a reference peak frequency of 170 Hz (±5 Hz). The LRA motors were directly tethered to a power supply with 0.1 mm diameter copper wire and fixed to the disk at the predetermined position with epoxy glue. We chose an electrically tethered design for simplicity and used very thin wires with a small amount of slack to minimize their effect on the motion of the disk. The robot disks were broadly divided into two shapes: circular disks with three vibration sources, and elliptical disks with two vibration sources (see Figure 1D). The design details of the adhesive disks specific to each experiment will be described below. Although our tethered, actuated disks were relatively simple, previous work has shown how power and communication could be added to make a remotely operated robot (Weston-Dawkes et al., 2021). Thus, in this work, for simplicity, we will refer to the actuated disk as a robotic disk.

#### 2.1.2 Signal generation

The signals that drive the LRA motors were generated by two synchronized signal generators (model SDG 2042X, SIGLENT), each with two output channels that control the frequency, peak-to-peak voltage (Vpp), and phase difference of the output signals (see Figure 1F). During the experimental tests, coordination between one or two function generators was required due to the different number of motors on the robotic disk.

#### 2.1.3 Testing platform

We used a flat surface to conduct the experiments. The surface was a smooth acrylic plate with labeled lines engraved on the back side to facilitate observation and data collection (Figure 1E). All experiments were performed on the smooth side. To prevent erroneous random events from being recorded and to minimize experimental error due to environmental factors, we performed at least three trials for each data point in all experiments. For each trial of each test, the robotic disk started its motion from the center of the plane, and its rotation angle, position displacement, and running time data were recorded.

### 2.2 Simplified model of robotic disks

In this section, we discuss a simplified model of a robotic disk traveling on the underside of a horizontal surface (see Figure 1C). In the illustrated free-body diagram, *F*_*g*_ = *mg* represents the gravitational force acting on the robotic assembly, which includes the plastic disk and multiple vibration motors. Since the robot is surrounded on all sides by air, its weight is balanced entirely by the contactless attraction force ${\bar{F}}_{\mathrm{adhesion}}$ induced by disk vibration, as described in § 1. Lateral asymmetry in the disk vibration gives rise to a propulsive force ${\bar{F}}_{p}$ that drives the disk along the indicated direction of motion, resulting in an opposing aerodynamic drag force ${\bar{F}}_{d}$ .

Each of the forces mentioned above, with the exception of *F*_*g*_, represents the time average of a corresponding unsteady force generated due to the disk vibrations. Provided that sufficient time has passed since the onset of stable adhesion to allow the decay of transient structural and aerodynamic phenomena, the dynamic system may be assumed to have achieved periodicity, whereby these aerodynamic forces may be approximated as Fourier expansions involving integer multiples of the applied vibration frequency *f* (Melikhov et al., 2016). For instance, the instantaneous propulsive force acting on the top surface of the disk may be expressed as

$$F_{p}\left(t\right)=\overset{\infty }{\underset{n=0}{\sum }}Re\left\{A_{p}^{\left(n\right)}e^{inft}\right\},$$ (1)

where $A_{p}^{\left(n\right)}$ for n∈Z≥0 represents the set of relevant Fourier coefficients, whence the steady propulsive force may be computed simply as the average value over one oscillation cycle

$${\bar{F}}_{p}=f\int_{0}^{1/f}F_{p}\left(t\right)\;dt=Re\left\{A_{p}^{\left(0\right)}\right\}.$$ (2)

Similar equations can be written for the steady drag ${\bar{F}}_{d}$ and the steady attractive pressure force ${\bar{F}}_{\mathrm{adhesion}}$ .

Of specific interest to us is the state of steady robotic translation, described by a balance of forces along the axis of motion, ${\bar{F}}_{p}={\bar{F}}_{d}$ . Note that the uniaxial LRA motors are mounted so as to provide negligible acceleration on the plane of motion—an assumption that was confirmed in our experiments, where the disk appeared to translate smoothly and exhibited an apparent ‘average’ drifting motion of characteristic speed $\dot{x}$ . For a specific robotic disk structure, the propulsive force depends on the voltage *U* applied to each motor and the phase shifts Δ*θ* between adjacent motors (for a model with *N* motors, Δ*θ* is an *N*-vector). The drag force depends on the translational speed (Anderson, 2016), as given by

$${\bar{F}}_{d}=\frac{1}{2}\rho {\dot{x}}^{2}\pi L^{2}C_{D}\left(\bar{Re}\right),\;with\;\bar{Re}=\frac{\rho \dot{x}L}{\mu },$$ (3)

where *ρ* and *μ* represent the density and dynamic viscosity of the ambient air surrounding the robot, *L* is a characteristic length associated with the disk planform, *C*_*D*_ is the collective drag coefficient of the robotic assembly and $\bar{Re}\approx 4$ is the relevant Reynolds number of steady locomotion, the latter estimated with the use of experimental data presented in the following sections. We may thus model the dependence of the transport speed on the applied voltage and phase shifts using the implicit relation

$${\dot{x}}^{2}\;C_{D}\left(\frac{\rho \dot{x}L}{\mu }\right)=\frac{2F_{p}\left(U,\Delta \theta \right)}{\rho \pi L^{2}}.$$ (4)

A similar relation can be readily developed for the rotational speed. In both cases, a complete, predictive model would require a rigorous solution of coupled partial differential equations describing the fluid-structure interactions between the deforming disk and the surrounding airflow. As a first exploration of this system, in the following sections, we empirically investigated the dependence of the propulsive force and the transport speed on the parameters *U* and Δ*θ* using two different representative robotic disk designs.

## 3 Results

### 3.1 Results of experiments using robotic disks with three sources of vibration

**FIGURE 2:**
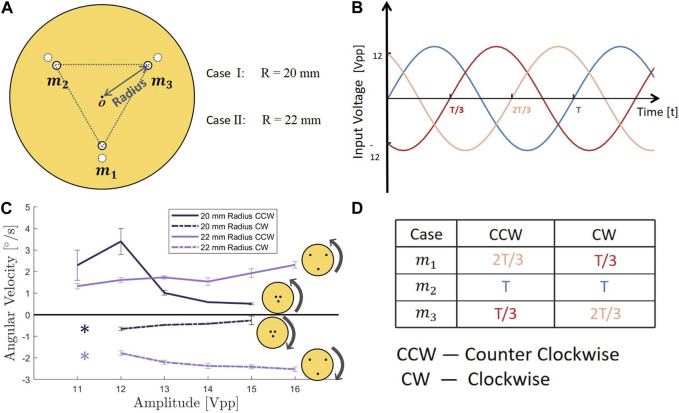
**(A)** Parameter ‘Radius’ shows the distance between the center of the circular robotic disk and three vibration motors. **(B)** Three input sinusoidal signals with 120-degree phase offsets. **(C)** Experimental results of three vibration sources model, relationship between input voltage amplitude and rotating angular velocity of two different radius sub-models. (Asterisks show no data at this point. The adhesive force of the robotic disk was too weak to stably stick to the surface.) (Error bars represent standard deviation of three trials.) **(D)** Relationship between the turning direction of robotic disk and the sequence of three 120-degree interval phase offsets.

To investigate the ability of a system capable of vibration adhesion to simultaneously generate controllable torques, we investigated a minimal design with three vibration sources (similar to designs used previously for acoustic levitation (Gabai et al., 2019)). We constructed a circular disk model with a radius of 80 mm equipped with three motors arranged in an equilateral triangle on the disk, such that the center of the triangle coincided with the center of the disk (see Figure 2A).

#### 3.1.1 Using a phase offset in the motion of the vibration sources to generate a torque

Previous work on acoustic levitation has generated torque applied to a levitated object using three vibration sources with a phase shift of 120° between each pair of sources, resulting in a traveling wave of vibration around the ring supporting the object (Gabai et al., 2019). By analogy, we hypothesized that we could use a similar pattern of vibration to generate torques for our adhesion robotic disk with three vibration sources. Furthermore, we expected that reversing the direction of the phase offset would reverse the direction of the torque. To investigate whether this approach could be used to rotate the robotic disk in a controllable direction, we conducted the following experiment: we placed the three-sources model on the experimental platform described above, and used the function generators to apply sinusoidal input signals with a frequency of 155 Hz, an amplitude of 12 Vpp, with a 120° phase offset to the three vibration sources, as shown in Figure 2B.

Our experimental results showed that for the counter-clockwise rotation case, the angular velocity was 3.4 ± 0.5°/s, whereas, in the clockwise rotation case, the angular velocity was −0.7 ± 0.1°/s. Both cases had relatively small linear motion (0.7 and 0.1 mm/s). There were notable differences in the angular velocities of the model in both directions, leading us to hypothesize that the cause of this phenomenon was asymmetry in the positions of the three motors on the disk. Previous work on acoustic levitation has shown that even small errors in the positions of vibration sources can have a significant impact on the resulting fluid motion (Ilssar et al., 2016; Ilssar and Bucher, 2017). To explore this hypothesis, we conducted further experiments which we describe in the section titled ‘4.2.2 The effect of asymmetry in the position of the vibration sources on the motion of the robotic disk’. Such results demonstrated that the 120° phase shifted vibration of three sources caused the disk to achieve rotation with very little translation. The modification of the sequence of three 120-degree interval phase offsets correspondingly altered the turning direction of the robotic disk. This relationship is visually illustrated in Figure 2D.

#### 3.1.2 Effect of amplitude on rotational velocity

After determining that the 120-degree interval setting enabled rotation of the robotic disk, we sought to investigate the effect of changing the amplitude of the input voltage on the resulting angular velocity. We conducted separate experiments for two different directions of 120° phase offsets and swept the input voltage from 11 Vpp to 16 Vpp with a 1 Vpp increment. The experimental results are shown in Figure 2C.

Surprisingly, we found that the absolute value of the angular velocity decreased as the input voltage increased, regardless of the direction of rotation, whether clockwise or counterclockwise. This indicated that increasing the input energy led to a slowdown in the rotation of the disk. We thus hypothesized that there was interference between the vibrations caused by each source, due to their proximity, which reduced the effectiveness of the torque generation.

#### 3.1.3 Effect of the radius of vibration sources on angular velocity

To validate our hypothesis about nearby vibration sources reducing the effectiveness of the torque generation, we tested vibration disks with greater spacing between the three motors. Specifically, we increased the circular distance between the motors and disks to 22 mm while maintaining all other settings and conducted the same experiment as before.

The results are presented in Figure 2C and conclusively confirm our assumption that as the input voltage increased, the absolute value of the angular velocity continued to increase once the radius reached 22 mm. Our findings suggested that the distance between the motors was a critical variable that impacted the operation of the robotic disk. When the motors were too close to each other, the three supposedly independent vibration sources could interfere with one another. Moreover, increasing the input energy might intensify this disturbance, leading to a reduction in output and performance. Furthermore, during the rotational motion, there were some small linear displacements of the disk, which theoretically should not have occurred. We hypothesize and argue for the possible reasons behind this unintended linear displacement in the Discussion section.

From the results presented above, we can infer the relationship between the input voltage and the rotational velocity of the three vibration sources robotic disk model under suitable motor spacing conditions. Within the tested voltage range (11–16 Vpp), the impact of the input voltage *U* on the clockwise and counterclockwise rotational velocities can be approximated as linear (with the corresponding coefficient of determination indicated in brackets):

$$\begin{array}{cc} CCW:\;\dot{\theta }\; & \approx \;0.16\;U+1.17\;\left(R^{2}=0.78\right)\\ CW:\;\dot{\theta }\; & \approx \;-0.17\;U-1.58\;\left(R^{2}=0.86\right) \end{array}$$ (5)

We also conducted experiments on how the robotic disk generates linear motion. For space reasons, please check (Supplementary Material text document) for the experimental results.

### 3.2 Results of experiments using robotic disks with two sources of vibration

**FIGURE 3:**
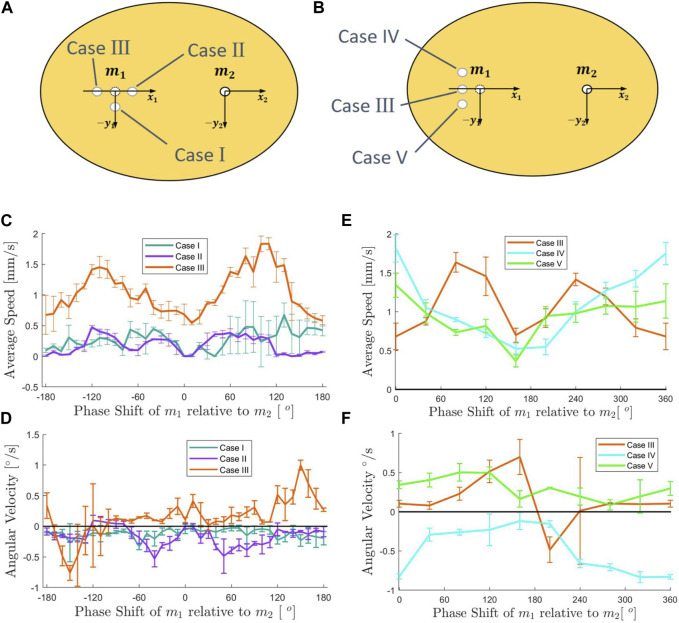
**(A)** Design of two vibration sources ellipses shape sub-model, Case I, II, III. **(B)** Design of two vibration sources ellipses shape sub-model, Case III, IV, V. **(C)** Experimental results for two vibration sources sub-models I, II, III. relationship between input signal phase shift and average linear speed. **(D)** Experimental results for two vibration sources sub-models I, II, III. Relationship between input signal phase shift and angular velocity. **(E)** Experimental results for two vibration sources sub-models III, IV, V. Relationship between input signal phase shift and average linear speed. **(F)** Experimental results for two vibration sources sub-models III, IV, V. Relationship between input signal phase shift and angular velocity. (Error bars represent standard deviation of three trials).

In the previous section, it was established that three vibration sources can be used to achieve rotational and linear motion of a disk-like robot in inverted surface conditions. To explore the possibility of reducing the number of vibration sources required for achieving similar effects, we designed a model consisting of an elliptical disk vibrated by two motors. The lengths of the major and minor axes of the oval were 160 mm and 140 mm, respectively. The optimal initial positions of the two motors were located at the two focal points of the ellipse on the major axis. However, placing both motors in the absolute focus position resulted in a completely symmetric state. Sharing ideas from a similar study (Rhee et al., 2022), the completely symmetrical shape would make the direction of motion undetermined under any energy input. Thus, a certain degree of asymmetry was introduced to achieve a controllable motion of the robotic disk. Three asymmetric sub-models were created based on the existing elliptical model. In each case, the position of one motor was kept at the focus point, while the other motor was intentionally moved off-focus, as shown in Figure 3A. The three cases were: Case I, where the off-focus motor was moved 1 mm downward from the original focus position; Case II, where the off-focus motor was moved 1 mm to the right; and Case III, where the off-focus motor was moved 1 mm to the left. The displacement distance was determined through experimental tests, as an off-focus distance that is too large would excessively destroy the overall symmetry and weaken the adhesion force.

#### 3.2.1 Controlling the motion of three sub-models with two motors on an inverted surface with a phase shift in the vibration

Based on the three existing sub-models for the three cases, we conducted experiments to investigate the effects of different relative phase shifts on the average linear speed and angular velocity of the robotic disk under the inverted surface condition. The input frequency of the two motors was fixed at 160 Hz, and the input voltage was set at 10 Vpp. We varied the relative phase shift between the two motors from 0° to 360° in increments of 10°, and recorded the running time, displacements in *x* and *y* directions, and overall turning angle. The average linear speed and angular velocity were calculated based on the collected data, as shown in Figures 3C, D.

The experimental results indicated that Case III outperforms the other two cases in terms of both average linear speed and angular velocities. Specifically, Case III exhibited significantly higher values of average linear speed and a wider range of thresholds for angular velocity. These findings suggested that introducing a small degree of asymmetry in the overall structure by moving one motor slightly away from the other motor could enhance the system’s performance. Our hypothesis was that adding asymmetry to the vibrational modes of the disk caused a net flow of air through the disk from one side to the other. We leave the testing of this hypothesis to future work. These results provided valuable insights into the design and optimization of vibration-based robotic systems with reduced numbers of vibration sources.

#### 3.2.2 The effect of asymmetry in the position of the vibration sources on the motion of the robotic disk

For the three sub-models of the two vibration sources models, it was found that the results of several experiments demonstrated stable and repeatable average linear speed and angular motions for Case II and Case III (see Figures 3C, D). However, this raised theoretical concerns. The overall structure of Case II and Case III should remain symmetric with respect to the *y*-axis, despite the fact that the vibration source, which should have been located at the focus of the ellipse, was shifted to the left and right relative to the *x*-axis. In theory, we should observe some large variation of certain data points of both average linear speed and angular velocity, in adjusting the phase shift from 0° to 360°. However, all the data showed nice consistency with no sign of huge fluctuations. We hypothesized that this phenomenon could be attributed to manufacturing deviations in the *y*-axis direction of the actual position of the motor, which was manually placed in the preset position by human hands. As a result, this manufacturing error created an undesired asymmetry.

To test our hypothesis about manufacturing asymmetries leading to the directionality of the angular velocities observed with the Case II and Case III models, we designed two additional sub-models, Case IV and Case V, based on Case III (as depicted in Figure 3B). These sub-models were positioned on the *x*-axis identical to Case III, but each was shifted by 1 mm with respect to the *y*-axis. By doing so, we were trying to determine if Case III exhibited any *y*-axis offset in actual situations. The experiment was then repeated for both average linear speed and angular velocities relative to phase shift, and the results are presented in Figures 3E, F.

Our experiments with Cases IV and V helped clarify the effects of vertical asymmetries on the ability of a model with two motors to achieve controllable linear and angular motions. Firstly, we were able to determine the *y*-axis asymmetry of Case III. Based on the angular velocity results, it was evident that both Case III and Case V had positive angular velocity when the phase shift was 0°, and the trend of the angular velocity curves was similar. Therefore, we concluded that Case III had a downward shift in the *y*-axis during the production process, resulting in undesired asymmetry. Additionally, we observed the effect of shifting the vibration source in the *y*-axis direction on the motion of the robotic disk. Case IV and Case V had nearly identical average linear speed profiles, while the average linear speed profile of Case III was significantly different from these two. With regard to the angular velocity data, although Case III and Case V were similar, Case III exhibited a sharp fluctuation at around 180-degree. We hypothesize that the robotic disk was in an “active” state when the vibration sources were located close to the structural symmetric position. This indicated that the control of the robotic disk using only two vibration sources became more challenging.

### 3.3 Verification of basic controllability

Through the experiments in the previous chapters, we have learned that we could adjust the input parameters to realize the motion of the robotic disk under inverted surface conditions. Our next goal was to realize the control of the robotic disk. Initial attempts at conducting tests under inverted surface conditions proved challenging, as alterations in the input parameters during motion frequently led to the robotic disk falling off the surface. Consequently, a fresh investigation was initiated under right-side-up surface conditions, where the robotic disk operated on the top side of a horizontal surface. This shift was motivated by the understanding that mastering control on a horizontal surface serves as a fundamental prerequisite for achieving control on an inverted surface. By adjusting the frequency of the input signals, we were able to achieve linear and rotational motions of the disk in various directions on right-side-up surfaces (see Supplementary Material text document). Similar to the experiments described above, we collected the displacement and rotation data of the robotic disk under different parameter settings over a specific period and analyzed the data.

**FIGURE 4:**
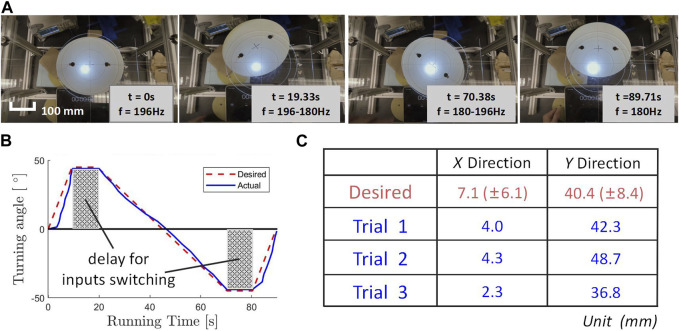
**(A)** Image from verification of basic controllability experiment, showing the motion of robotic disk. **(B)** Desired and actual trajectory of turning angle *versus* running time. (The gray area represents the time when we switched the input parameters and the robotic disk was having a delay.) **(C)** Robotic disk final landing location, calculated and experimental results.

To prove that the data collected in our experiments could indeed be used to control the robotic disk, we conducted a verification experiment. We predefined the target endpoint and motion trajectory of the robotic disk and controlled the disk to move according to the predefined route by adjusting the input parameters. We designed a task for the robotic disk to move to a target point on the plane. While we are currently unable to completely isolate displacement and rotation, we were able to combine motion trajectories generated by different parameter settings to achieve a desired position, resulting in an overall linear motion. We chose to conduct this experiment in the right-side-up surface condition and programmed the robotic disk to rotate 45° counterclockwise at 196 Hz, followed by a 90° clockwise rotation at 180 Hz, and finally a 45° counterclockwise rotation again at 196 Hz (see Figure 4A). The whole moving process was divided into three stages, each of which could be regarded as the rotation plus displacement motion of the robotic disk in the 2D plane. Using members of the planar Special Euclidean group SE(2) to represent these three motion segments, the whole process can be written using three 3 × 3 homogeneous transformation matrices *T*_1,2,3_:

$$T_{1,2,3}=\left[\begin{array}{cc} R_{1,2,3} & p_{1,2,3}\\ 0 & 1 \end{array}\right]\;\;R\in SO\left(2\right),\;p\in R^{2}$$ (6)

where *R* is the rotation matrix and *p* is the vectors representing the origin of the robotic disk body in the space frame. From the data we collected in previous experiments, we have:

$$\begin{array}{ccc} R_{1}=R_{3} & =\left[\begin{array}{cc} \cos\left(\pi /4\right) & -\sin\left(\pi /4\right)\\ \sin\left(\pi /4\right) & \cos\left(\pi /4\right) \end{array}\right]\;p_{1}=p_{3} & =\left[\begin{array}{c} -16.61\\ 47.24 \end{array}\right]\\ R_{2} & =\left[\begin{array}{cc} \cos\left(\pi /2\right) & -\sin\left(\pi /2\right)\\ \sin\left(\pi /2\right) & \cos\left(\pi /2\right) \end{array}\right]\;\;p_{2} & =\left[\begin{array}{c} -35.38\\ -38.21 \end{array}\right] \end{array}$$ (7)

Therefore, the calculated final configuration of the robotic disk would be:

$$\begin{array}{ll} T_{\mathrm{final}} & =T_{1}T_{2}T_{3}\\ & =\left[\begin{array}{ccc} \cos\left(\pi /4\right) & -\sin\left(\pi /4\right) & -16.61\\ \sin\left(\pi /4\right) & \cos\left(\pi /4\right) & 47.24\\ 0 & 0 & 1 \end{array}\right]\\ & \;\times \left[\begin{array}{ccc} \cos\left(\pi /2\right) & -\sin\left(\pi /2\right) & -35.38\\ \sin\left(\pi /2\right) & \cos\left(\pi /2\right) & -38.21\\ 0 & 0 & 1 \end{array}\right]\\ & \;\times \left[\begin{array}{ccc} \cos\left(\pi /4\right) & -\sin\left(\pi /4\right) & -16.61\\ \sin\left(\pi /4\right) & \cos\left(\pi /4\right) & 47.24\\ 0 & 0 & 1 \end{array}\right]\\ & \approx \left[\begin{array}{ccc} \;1 & \;0 & 7.1\\ \;0 & \;1 & 40.4\\ \;0 & \;0 & 1 \end{array}\right] \end{array}$$ (8)

This meant that the total rotation of the robotic disk should be zero. And taking into account the variation of the original three sets of trials at each data point, the expected variation (within one standard deviation) of the final result was (*x*, *y*) = (7.1 ± 6.1 mm, 40.4 ± 8.4 mm). The actual experimental results of landing locations are shown in Figure 4C.

Using image recognition software, the actual turning angle of the robotic disk and the expected curve were plotted. As shown in Figure 4B, the robotic disk closely followed the angular trajectory that was pre-set, with the grey portion representing the interval time for manually adjusting the input parameters. The final locations obtained from the three experiments were closely aligned with the expected landing location. While some error in the accuracy of the turning angle was observed, the magnitude of the error remained within the acceptable range. Thus, the experiment successfully verified that we could utilize the data we collected to control the motion of the robotic disk with an open-loop controlling method.

## 4 Discussion

### 4.1 Future work

There are multiple issues that require further investigation and are beyond the scope of this paper. Firstly, manufacturing errors caused unintentional asymmetry. In the ideal case, the model with three motors would be able to generate purely rotational motion (without linear displacement). However, even with rotationally symmetric geometric parameters and control inputs, a small amount of linear displacement existed in the actual experimental results. As described in the previous sections, the motors were manually affixed to the pre-engraved and marked positions on the disk by hand, a process that created human errors beyond our control. Although this error was very small, possibly as little as 0.01 mm, it had a significant impact on the performance of the robotic disk. According to a study by (Minikes et al., 2005) on the generation of traveling waves in rigid materials, even a small deviation of the vibration source from the optimal position could significantly affect the shape of the traveling wave and reduce the force exerted on the object. However, this does not affect the conclusions drawn from our experiments. According to what we learned from the further exploration of elliptical model structural asymmetry experiments, although unintentional asymmetry might cause shifts in the data points, the trend in the effect of different parameters on robotic disk motion remained stable and constant. To control this unstable system, other suitable methods, such as feedback control, could be introduced. Such a method could help decouple rotational and linear motion, as well as compensate for the misplacement of the actuators and other uncertainties.

Secondly, the LRA motors we are using in this design caused the robotic disk to have a lower loading capacity. In our previous study, we demonstrated the load capacity using the adhesion force generated by a single ERM motor vibration. However, the load capacity of this robotic disk was reduced. This result can be attributed to the different operating mechanisms of the two motors. With the same voltage rating, ERM motors can provide relatively high vibration amplitude output, but they cannot control the phase shift between motors; in contrast, LRA motors can control the phase shift between motors, but their output vibration is relatively weak (Poyraz and Tamer, 2019). We attempted to find a higher output LRAs product on the market, but we could not find one that is available for purchase. Exploring the design of custom LRA motors could enhance the vibration power, thereby improving the overall motion capabilities of the system. Additionally, investigating the feasibility of combining multiple robotic disks together could be explored to increase the system’s load capacity and expand its potential applications.

Another avenue for future work involves the construction of a general mathematical model for the vibration adhesion method. While this paper presented a series of experimental data highlighting the impact of input voltage and phase shift on the motion performance of the disk model, there are other variable parameters, such as the position of motors, input frequency, disk dimensions, and material thickness, that were not extensively explored in this study. Developing a comprehensive mathematical model could be beneficial in understanding and predicting the behavior of the robotic disk under various parameter combinations and conditions.

Additionally, to enhance the accuracy and reliability of the mathematical model, the development of non-invasive measurement techniques to precisely capture the oscillation waveform of the disk would be advantageous. Utilizing non-invasive methods, such as laser-equipped devices, would enable researchers to characterize the systems without affecting the exact flexing motion. Accurate characterization of the oscillation waveform could contribute to the validation and refinement of the mathematical model, leading to a deeper understanding of the underlying principles governing the motion of such a robotic disk.

### 4.2 Conclusion

In summary, this paper introduced a novel approach utilizing multiple vibration sources to generate adhesive and normal forces. A robotic disk model with flexible plastic material was constructed, enabling rotation and displacement solely through vibration adhesion without the need for an assisting system. Notably, this study represents the first known instance of conducting adhesive torque generation experiments. By means of a series of experiments, the feasibility of driving the robotic disk through vibration adhesion force was verified, and several key parameters were identified that control robotic disk motion and exhibit a tendency to influence it. This work represents a step towards the construction of a small-sized tetherless robot that can overcome gravity and move freely in a general environment. This method has great potential for applications such as high-altitude cleaning operations and special environmental monitoring.

## Data Availability

The raw data supporting the conclusion of this article will be made available by the authors, without undue reservation.
