# Supplementary material for: Controlling the motion of gas-lubricated adhesive disks using multiple vibration sources
Source: Front Robot AI. 2023 Oct 30;10:1231976. doi: 10.3389/frobt.2023.1231976 (PMC10642911; doi:10.3389/frobt.2023.1231976)
Supplement: Supplementary file 1 [file DataSheet1.PDF]

Supplementary Material

1 LINEAR MOTION GENERATION USING DISKS MODEL WITH THREE VIBRATION SOURCES

1.1 Can a linear phase shift be used to generate linear velocity?

After successfully generating torque with the circular model using three motors, we attempted to generate linear displacement by adjusting the motor settings. Our first attempt involved modifying the relative phase differences between the three motors, trying to achieve linear motion in a specific direction by altering the shape of the internal traveling wave. We set the input frequency of the three motors to 155 Hz and the input voltage to 12 Vpp. We synchronized the running phase of two motors and used it as a reference zero to adjust the relative phase difference of the remaining motor. This configuration resulted in a bilateral-symmetrical structure with a straight line from the disc's center to the motor set differently (see Fig S1.A). During the experiment, we varied the relative phase shift of the motor in 30-degree increments, ranging from 0 degrees to 360 degrees. The initial position of the disk's center was located at the origin of the coordinate system. Motors 2 and 3 were positioned in the positive y-axis direction, while motor 1 was situated on the negative y-axis. The results are shown in Figure S1.B and C.

The experimental results indicated that the average linear speed and angular velocity of both the 20 mm and 22 mm radius sub-models were correlated with the phase shift of the motor. The angular velocity curves of the two sub-models had similar sinusoidal trends, with high negative values at 0 degree phase shift and high positive values at approximately 210 degree phase shift. In terms of average linear speed, the 22 mm radius sub-model exhibited a wider range of variation, with high values at 0 degree phase shift and lower values at 180 degree phase shift. Meanwhile, the 20 mm radius sub-model had a smoother variation in average linear speed, with lower values also observed at 180 degree phase shifts. The experimental outcomes suggested that for each sub-model, there existed two phase shift settings that resulted in zero angular velocity. Notably, the collected data from the 22 mm sub-model demonstrated that the 150 degree phase shift data point was located in proximity to zero angular velocity, and the actual motion trajectory was depicted in figure S1.E. Despite the motion of the disk motion not being precisely aligned with the bilateral symmetry axis, we were able to generate pure linear motion by adjusting the phase shift between motors.

Note that for simplicity, throughout this work we calculated the average linear speed based on the final location of the disk after a certain period of motion. During our experiments, the robotic disk mostly carried out arc trajectory motion, as shown in figure S1.D and E. However, calculating the average instantaneous linear velocity for these arcs was challenging, and only a few motions exhibited a small reversal in the travel direction. The reversal in travel direction was observed specifically in the 20 mm radius sub-model when the input phase offset was sweeping from 90-degree to 150-degree. Therefore, to simplify data collection, we calculate the average linear speed over the duration of an experiment using the initial and final positions of the disk. Assuming that the coordinates of the final position of the disk were (x, y), its average linear speed was calculated as $\sqrt{x^2 + y^2}$. Such a method in most cases represented a good approximation of the average instantaneous linear velocity.

1.2 Can the linear velocity be increased by adjusting the amplitude?

In continuation of the bilateral-symmetrical design approach, we sought to investigate the influence of the input voltage on the linear motion of the robotic disk (see Fig S1.F). To achieve this, we identified the phase shift settings for two sub-models with zero angular velocity, 178 degree for the 20 mm radius sub-model and 170 degree for the 22 mm radius sub-model, based on existing data. With the input frequency of all three motors still locked at 155 Hz, we maintained the fixed input voltage of the two relative phase synergistic motors at 12 Vpp, and swept the input voltage of the remaining motor with different relative phases from 10 Vpp to 15 Vpp, with a gap of 1 Vpp (see Figure S1.G and H).

From the experimental results, we found that the average linear speed of the two sub-model disks generally increased with an increase in the input voltage. For the 22 mm radius sub-model, this upward trend was not limited within the tested range. For the 20 mm radius sub-model, we conducted the same results as in the previous experiment. When the input voltage was too large, the motors interacted with each other, causing the average linear speed to decrease. However, as the input voltage changed, the angular velocity of the robotic disk did not remain at zero but fluctuated. For the 20 mm radius sub-model, the angular velocity changed less at 10-12 Vpp and stayed near zero, but when the input voltage increased to 13 Vpp or more, there was a significant positive change in the angular velocity. Similarly, the sub-model with a 22 mm radius stayed near zero at an input voltage of 11-14 Vpp, but there was a significant increase in angular velocity as the input voltage continued to increase. This meant that increasing the input voltage did increase the linear speed of the disk with the right spacing between motors. But the angular velocity did not remain zero as the voltage changed. The motion of the robotic disk profile was a mixture of rotational and linear arc trajectories.

2 RIGHT-SIDE-UP SURFACE RUNNING USING DISKS WITH TWO VIBRATION SOURCES

2.1 Can the robot work under right-side-up surface conditions?

In the previous section, we discussed the experimental results of the robot's motion under inverted surface conditions, where the robot was required to generate adsorption forces greater than its weight and driving forces parallel to the plane of motion while overcoming gravity. This is a challenging task compared to typical robot motion. Our goal in this section is to explore whether the vibration-based driving source and disk-like robot design can be effective under right-side-up surface conditions. We intend to investigate the necessary parameter adjustments and the impact of other input parameters on the robot's motion performance. Experimental trials were conducted with a frequency similar to previous experiments, but the results showed that the robot remained nearly immobile with no apparent displacement or rotation. This observation is consistent with previous study, which demonstrated that hard materials experience a small adsorption force at low frequencies that gradually increases to a thrust force as the frequency rises. Consequently, we raised the input frequency to 162 Hz, and successfully observed the motion of the robot disk.

2.2 How does frequency affect the motion of robot under right-side-up surface conditions?

We carried out an experimental investigation to study the impact of vibration frequency on a flexible plastic material disk robot. The sub-model used for testing was Case III, and the input voltage of the two motors was kept constant at 16 Vpp. The input frequency was swept from 160 Hz to 202 Hz with a 2 Hz

gap to examine the effect of vibration frequency on the performance of the robot. The experimental results are illustrated in Figure S2.B.

Results showed that input signal frequency affected the motion of the robot disk. The negative directional angular velocity of the robot increased continuously as the frequency increased from 160Hz to 184 Hz, reaching its maximum speed of clockwise rotation at 184 Hz. Thereafter, as the frequency increased from 184Hz to 198 Hz, the negative angular velocity decreased continuously, reaching zero around 192 Hz. The positive angular velocity then emerged and rose continuously, achieving the maximum speed of counterclockwise rotation at 198 Hz. Finally, as the frequency further increased from 198 Hz, the robot reached a stage where there was almost no displacement or rotation. The robot's frequency response under right-side-up surface conditions became a critical input variable that significantly affected the robot's torque generation, unlike the optimal frequency of operation under inverted surface conditions. Therefore, it can be inferred that the frequency of vibration needs to be adjusted based on the surface conditions to achieve optimal robot performance.

2.3 How does phase shift affect motion under right-side-up surface conditions?

In the case of inverted surface conditions, the relative phase shift of the two motors is one of the significant factors that control the angular velocity. However, to examine the extent of its effect on the angular velocity under right-side-up surface conditions, we carried out additional experiments. We selected two vibration frequencies, 184 Hz and 196 Hz, to represent the two directions of rotation of the robot, respectively. We kept the input voltage and the respective input frequency constant and swept the relative phase shift between the two motors from 0 degrees to 360 degrees, with a 40-degree increment. The experimental results shown in Figure S2.C indicate that the phase shift still has an effect on the robot's angular velocity, causing it to fluctuate within a certain range. However, the effect of the phase shift on the angular velocity is limited compared to the effect of frequency, as evidenced by the smaller range of fluctuations in the angular velocity.

3 SUPPLEMENTARY VIDEOS

Please check [Supplementary Video 01] and [Supplementary Video 02] attached to this paper.

4 SUPPLEMENTARY FIGURES

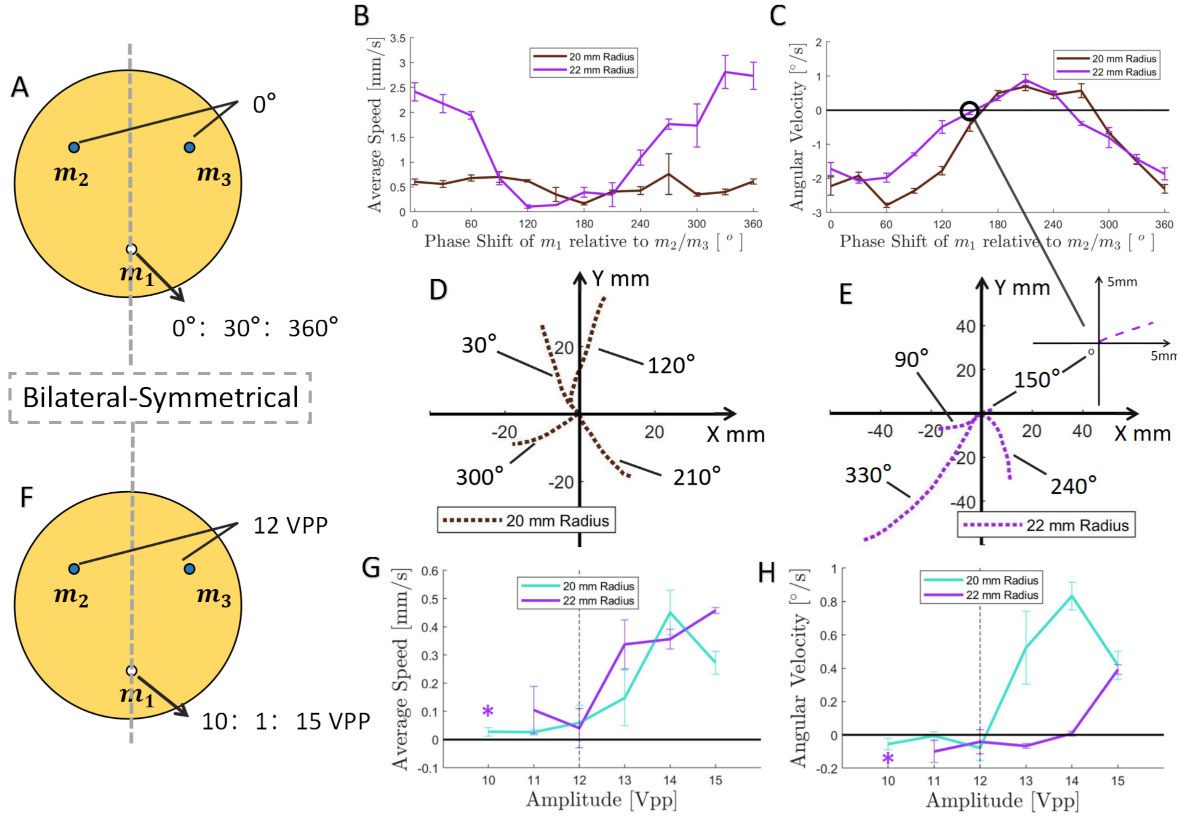

Figure S1. **(A)** Bilateral-symmetrical parameters setting for linear phase shift experiments. **(B)** Experimental result of relationship between phase shift and average linear speed. **(C)** Experimental result of relationship between phase shift and angular velocity, with a trajectory of motion when the angular velocity is zero. **(D)** Linear phase shift experiments, actual running trajectories of 20 mm radius sub-model. **(E)** Linear phase shift experiments, actual running trajectories of 22 mm radius sub-model. **(F)** Bilateral-symmetrical parameters setting for adjusting input voltage amplitude experiments. **(G)** Experimental result of relationship between input voltage amplitude and average linear speed. **(H)** Experimental result of relationship between input voltage amplitude and angular velocity. (Error bars represent standard deviation of three trials.)

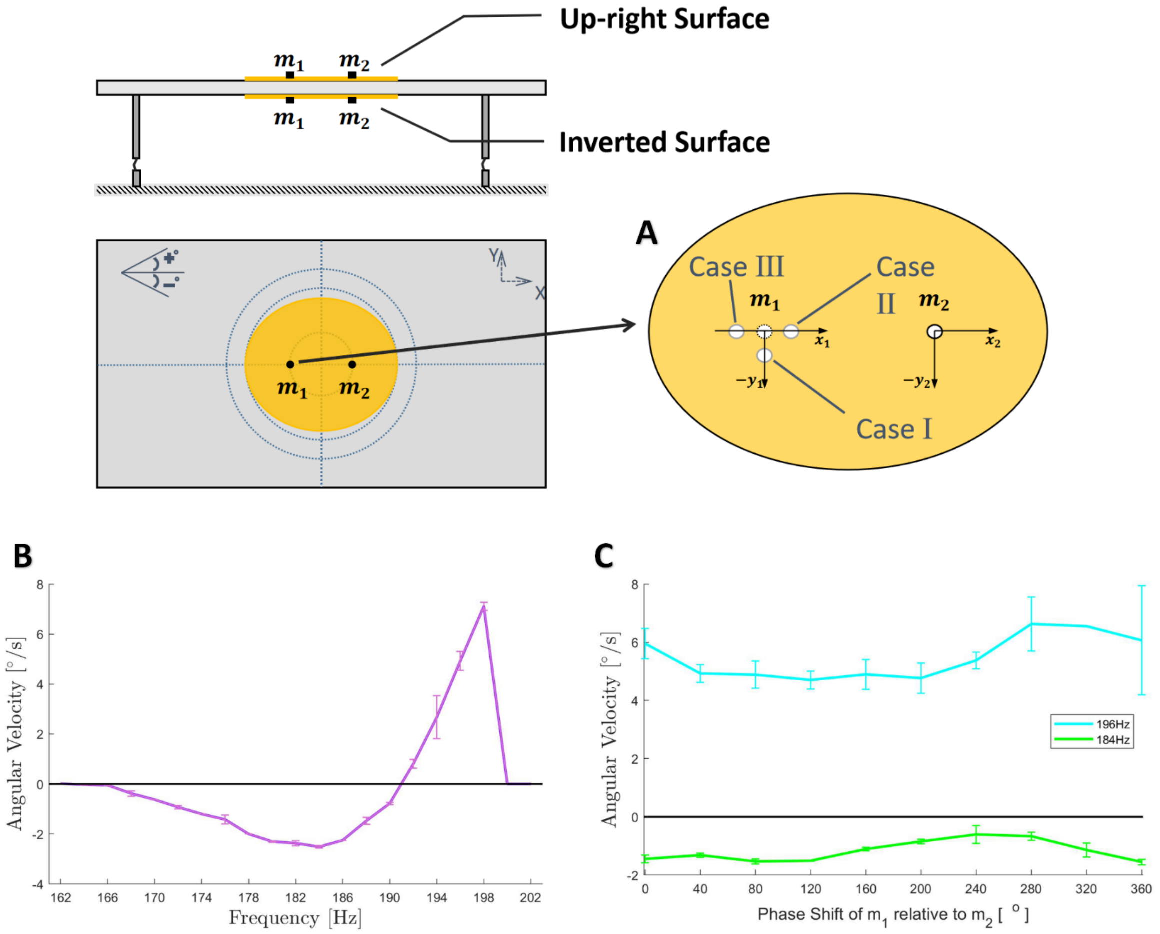

Figure S2. **(A)** Using Case III as the experimental sub-model for right-side-up surface condition testings. **(B)** Experimental results for two vibration sources right-side-up surface condition, frequency versus angular velocity testing. **(C)** Experimental results for two vibration sources right-side-up surface condition testing, phase shift versus angular velocity of two selected frequency cases. (Error bars represent standard deviation of three trials.)
